# Supplementary material for: Midbrain extracellular matrix and microglia are associated with cognition in aging mice
Source: Nat Commun. 2025 Nov 27;16:11319. doi: 10.1038/s41467-025-66434-z (PMC12722224; doi:10.1038/s41467-025-66434-z)
Supplement: Supplementary file 1 — Supplementary Information [file 41467_2025_66434_MOESM1_ESM.pdf]

Supplementary information for:

Midbrain extracellular matrix and microglia are associated with cognition in aging mice

Authors: Daniel T. Gray[1], Abigail Gutierrez[1], Yasaman Jami- Alahmadi[2], Vijaya Pandey[2], Lin Pan[3], Ye Zhang[3], James A. Wohlschlegel[2], Ross A. McDevitt[4], Lindsay M. De Biase[1]

Affiliations:

1. Department of Physiology, David Geffen School of Medicine, University of California, Los Angeles. Los Angeles, CA 90095
2. Department of Biological Chemistry, David Geffen School of Medicine, University of California, Los Angeles. Los Angeles, CA 90095
3. Department of Psychiatry and Biobehavioral Sciences, Semel Institute for Neuroscience and Human Behavior, David Geffen School of Medicine, University of California, Los Angeles. Los Angeles, CA 90095
4. Comparative Medicine Section, National Institute on Aging, Baltimore, MD, United States of America

Co-Corresponding Authors: Daniel T. Gray and Lindsay M. De Biase
Department of Physiology, David Geffen School of Medicine
University of California, Los Angeles
10833 Le Conte Avenue, 76-100E CHS; Los Angeles, CA 90095
E-mail address: dtgray@mednet.ucla.edu; lmdebiase@mednet.ucla.edu

Supplementary Figures:

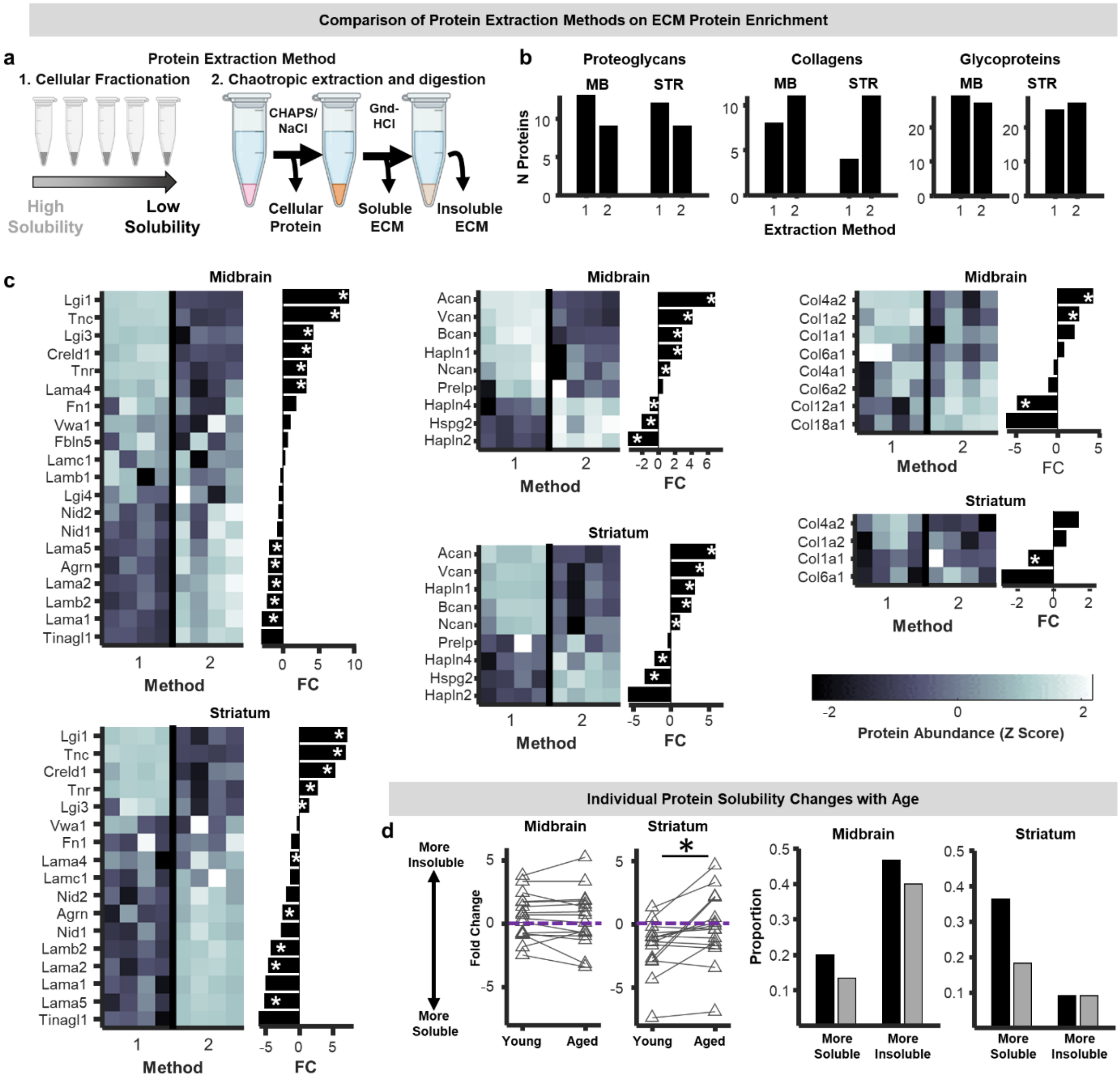

Supplementary Figure 1. Comparison of extracellular matrix enrichment strategies. **a)** The midbrain and striatum of young-adult (3 months) mice were dissected and underwent either 1: solubility-based subcellular fractionation (n=4 mice) or 2: chaotropic extraction and digestion (n = 4 mice) protocols. Schematic created in BioRender. Gray, D. (2025) https://BioRender.com/nq9fgld. **b)** Comparison of the number of core matrisome proteins detected using each approach. Extraction method 1 refers to solubility-based fractionation and extraction method 2 refers to chaotropic extraction and digestion **c)** Heat maps of protein abundances (z scored) and bar plots of corresponding fold-changes with age for ECM glycoproteins, proteoglycans, collagens, and ECM regulators detected using the two approaches. (* p < 0.05 - unpaired t-test). **d)** Left: Paired plots of fold changes of individual matrisome proteins detected in the insoluble fraction that were detected in at least one of the subcellular/soluble fractions using the fractionation approach for young and aged mice. Fold-changes were calculated with respect to the insoluble fraction. Purple lines denote a fold change of 0, indicating no difference in abundance between insoluble and soluble fractions. Right: bar plot showing the proportion of matrisome proteins showing differences in solubility (upregulated and downregulated shown separately) in the young and aged mice. Source data are provided in the file **Source Data - Figure S1.** * denotes p<0.05.

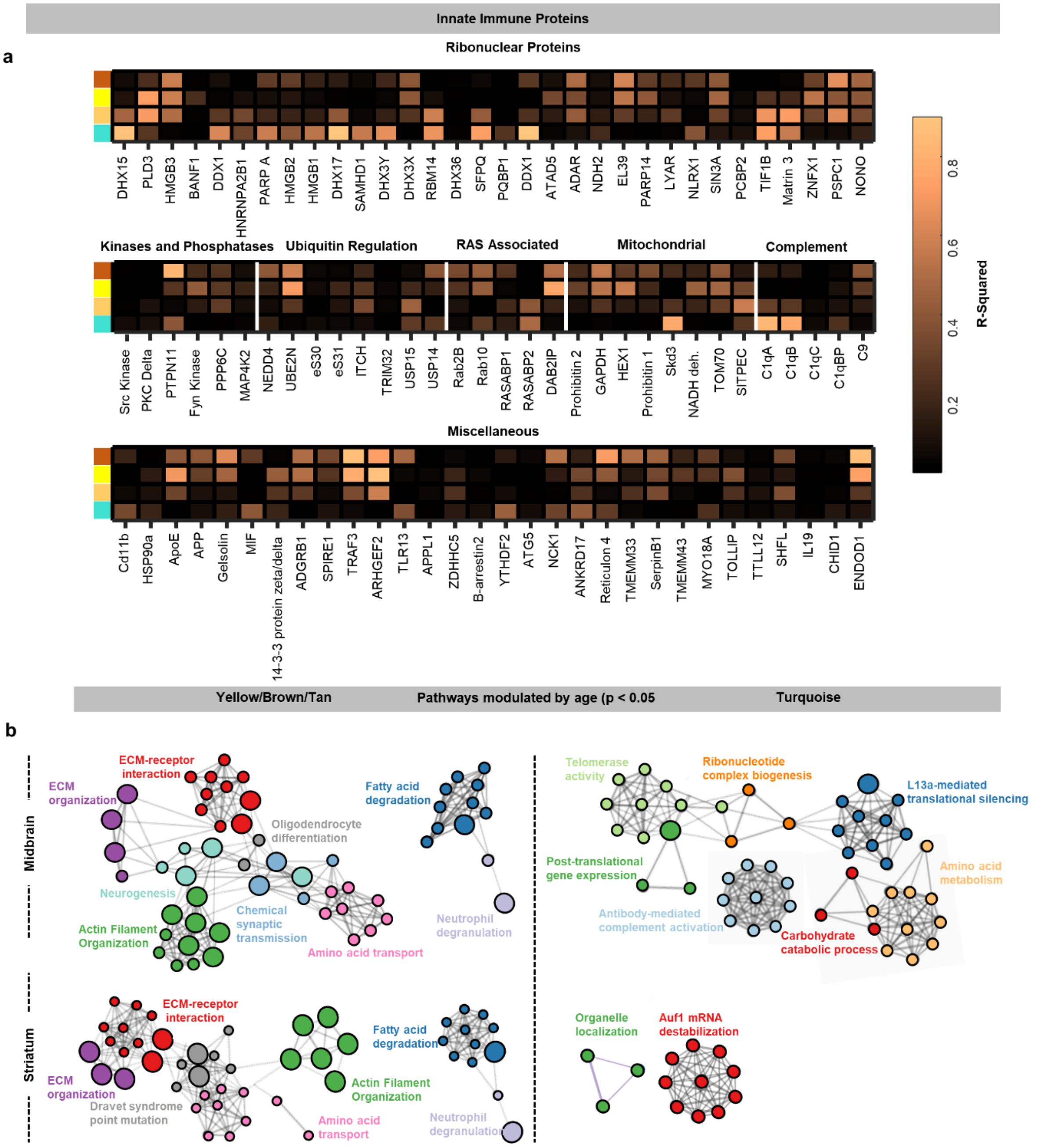

Supplementary Figure 2. WGCNA module-trait relationships of immune proteins and pathway analysis. **a)** Heatmaps representing correlations (r-squared values) between brown, yellow, tan, and turquoise module eigengenes and abundances of specific innate immune proteins separated by functional classification (ribonuclear proteins, kinases and phosphatases, ubiquitin-associated proteins, mitochondrial proteins, complement proteins, and other miscellaneous innate immune proteins). **b)** Network plots of process and pathway enrichment terms associated with all tan/yellow/brown module (left) and turquoise module (right) proteins whose abundances were modulated by age (p<0.05) in the midbrain and striatum. Data are derived from midbrain and striatum tissue from n=4 young mice (3-4 months) and n=4 aged mice (20+ months). Source data are provided in the file **Source Data - Figure S2.**

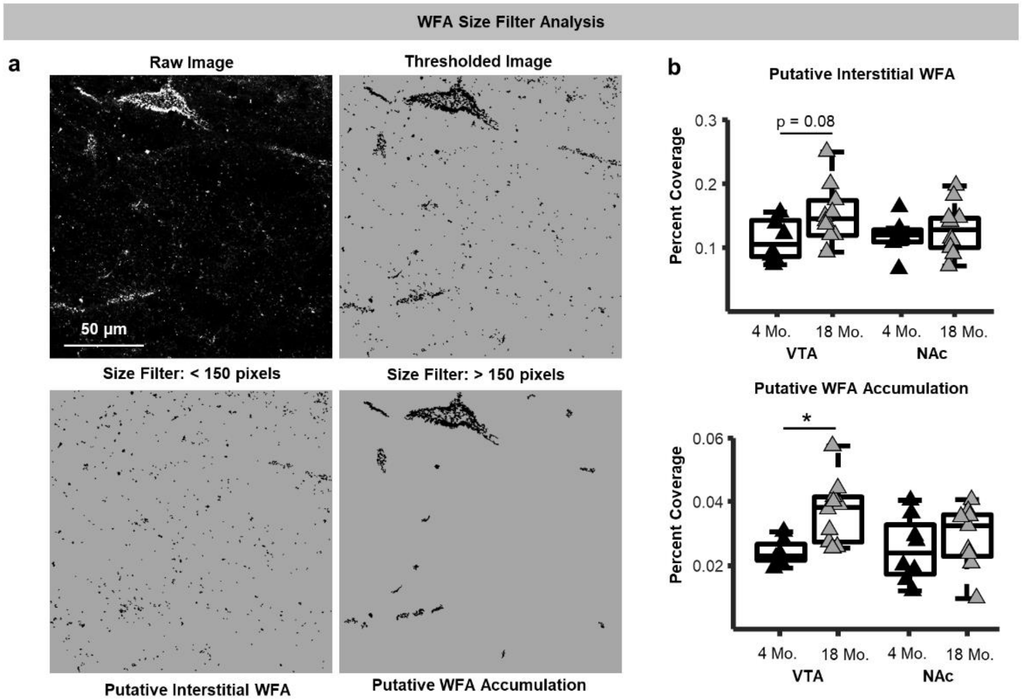

Supplementary Figure 3. WFA size filter analysis. **a)** Depiction of analysis used to segment WFA signal associated with the putative interstitial matrix from WFA signal associated with larger accumulations around neurons or vasculature. Raw images were thresholded and WFA puncta were size filtered with a cutoff value of 150 pixels. WFA puncta smaller than 150 pixels were classified as putative interstitial WFA and those larger than 150 pixels were classified as putative WFA accumulations. **b)** Top: boxplots of WFA tissue coverage from only putative interstitial WFA in the VTA and NAc of young-adult (black; n=8 mice; n=10 mice) and late-middle-aged (grey) mice. Bottom: boxplots of tissue coverage of putative WFA accumulations. Boxes represent the interquartile range (IQR; 25-75 percentiles), the middle line represents the median, and whiskers extend +/- 1.5*IQR. Source data are provided in the file **Source Data - Figure S3.** * denotes $p<0.05$.

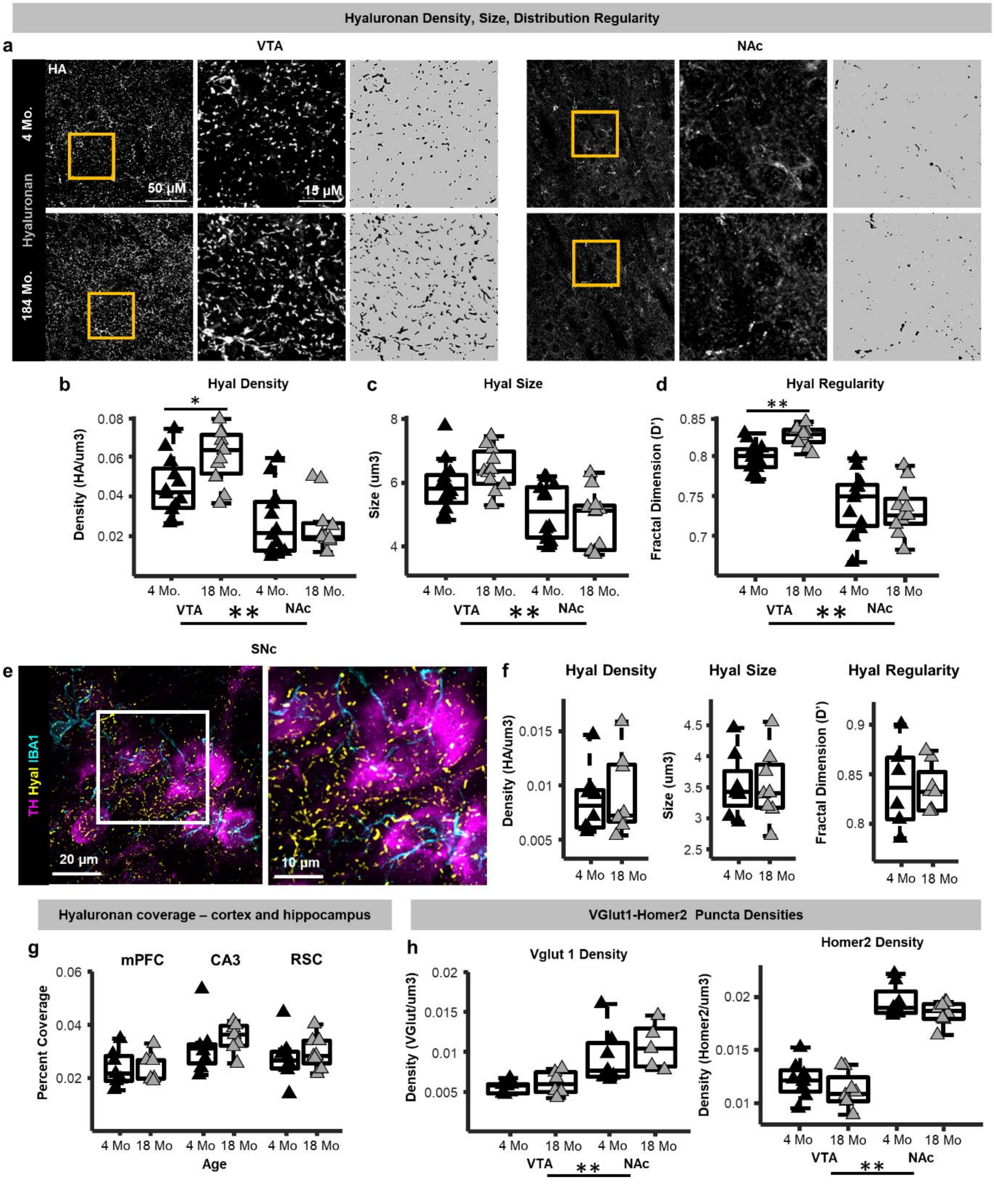

Supplementary Figure 4. Hyaluronan and synapse abundance across regions. **a)** The ECM scaffold hyaluronan (Hyal) was analyzed in the ventral tegmental area (VTA) and nucleus accumbens (NAc). **b)** Photomicrographs of histochemically labelled hyaluronan in the VTA and NAc of a young-adult (4 months; n=12 mice) and late-middle-aged (18 months; n=12 mice) mouse (left panels). Middle panels show zoomed in images of the areas within the yellow squares in the left panels. Right panels depict binarized images of the hyaluronan matrix used for quantification. **c-e)** Boxplots depicting **c)** hyaluronan fibril densities, **d)** median hyaluronan fibril sizes, and **e)** hyaluronan distribution regularity (Fractal Dimension plugin – Fiji) in the VTA and NAc of young-adult (black) and late-middle-aged (grey) mice. **e)** Example

photomicrograpghs of histochemically labelled hyaluronan, tyrosine hydroxylase (TH)-positive dopaminergic neurons, and IBA1-positive microglia in the substantia nigra pars compacta (SNc). **f**) SNc hyaluronan fibril densities, sizes, and distribution regularity were not different between young-adult (4 months; n=7 mice) and late-middle-aged mice (18 months; n=8 mice). **g)** Hyaluronan fibril field of view coverage in the mPFC, CA3 region of the hippocampus, and retrosplenial cortex (RSC) of young-adult (4 months; n=8 mice) and late-middle-aged mice (18 months; n=8 mice). **h)** Left: densities of VGlut1 puncta in the VTA and NAc of young-adult and middle-aged mice. Right: densities of Homer2 puncta in the VTA and NAc of young-adult and middle-aged mice. In all boxplots, boxes represent the interquartile range (IQR; 25-75 percentiles), middle lines the median, and whiskers extend +/- 1.5*IQR. Source data are provided in the file **Source Data - Figure S4.** * denotes $p<0.05$; ** denotes $p<0.01$.

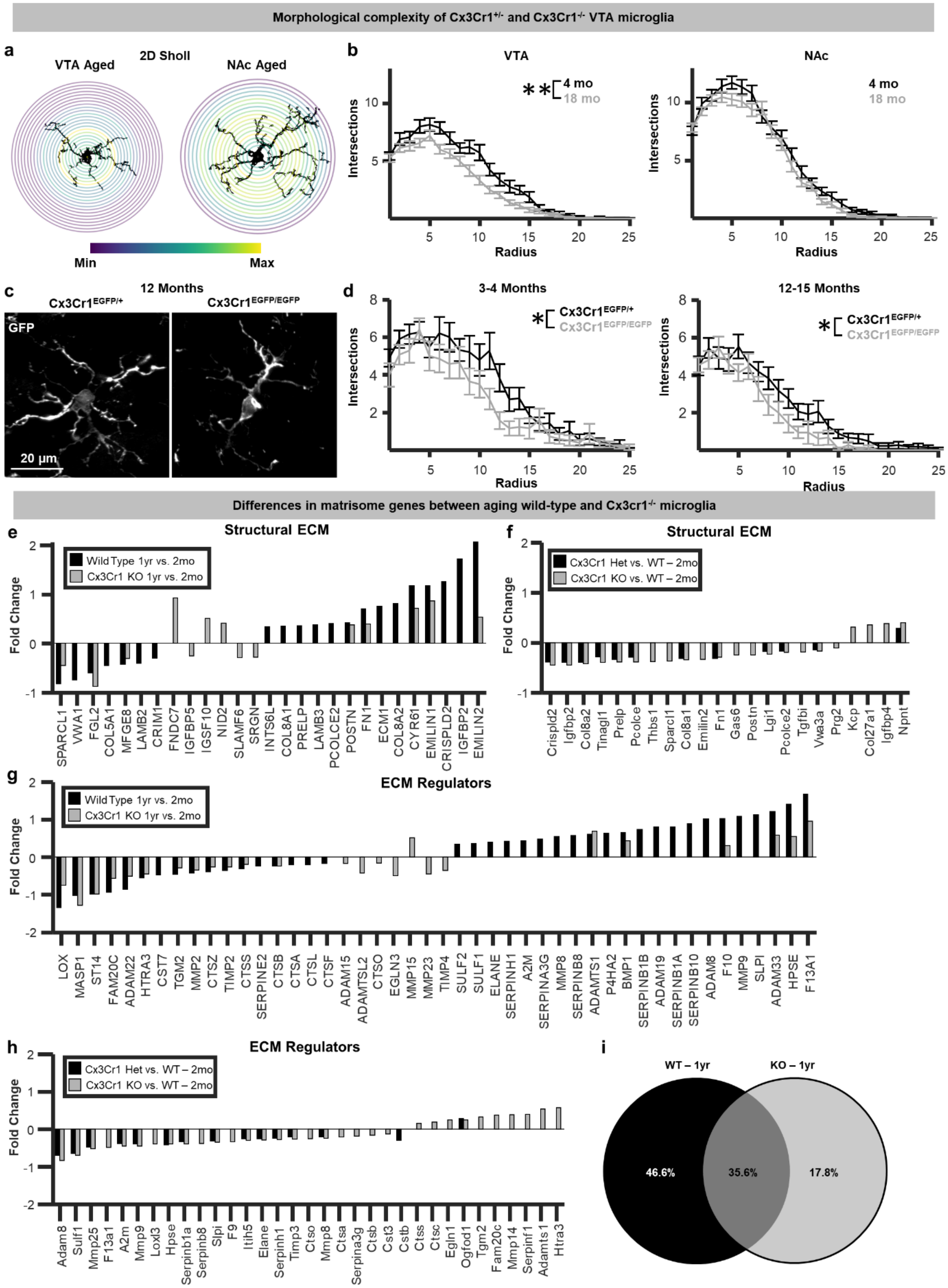

Morphological complexity of Cx3Cr1+/- and Cx3Cr1-/- VTA microglia
a
2D Sholl
VTA Aged
NAc Aged
Min
Max
b
VTA
NAc
4 mo
18 mo
Intersections
Radius
c
12 Months
Cx3Cr1EGFP/+
Cx3Cr1EGFP/EGFP
GFP
20 µm
d
3-4 Months
12-15 Months
Cx3Cr1EGFP/+
Cx3Cr1EGFP/EGFP
Intersections
Radius
Differences in matrisome genes between aging wild-type and Cx3cr1-/- microglia
e
Structural ECM
Wild Type 1yr vs. 2mo
Cx3Cr1 KO 1yr vs. 2mo
Fold Change
SPARCL1 VWA1 FGL2 COL5A1 MFGE8 LAMB2 CRIM1 FNDC7 IGFBP5 IGSF10 NID2 SLAMF6 SRGN INTS6L COL8A1 PRELP LAMB3 PCOLCE2 POSTN FN1 ECM1 COL8A2 CYR61 EMILIN1 CRISPLD2 IGFBP2 EMILIN2
f
Structural ECM
Cx3Cr1 Het vs. WT – 2mo
Cx3Cr1 KO vs. WT – 2mo
Fold Change
Crispld2 Igfbp2 Col8a2 Tinagl1 Prelp Pcolce Thbs1 Sparcl1 Col8a1 Emilin2 Fn1 Gas6 Postn Lgi1 Pcolce2 Tgfbi Vwa3a Prg2 Kcp Col27a1 Igfbp4 Npnt
g
ECM Regulators
Wild Type 1yr vs. 2mo
Cx3Cr1 KO 1yr vs. 2mo
Fold Change
LOX MASP1 ST14 FAM20C ADAM22 HTRA3 CST7 TGM2 MMP2 CTSZ TIMP2 CTSS SERPINE2 CTSB CTSA CTSL CTSF ADAM15 ADAMTSL2 CTSO EGLN3 MMP15 MMP23 TIMP4 SULF2 SULF1 ELANE SERPINH1 A2M SERPINA3G MMP8 SERPINB8 ADAMTS1 P4HA2 BMP1 SERPINB1B ADAM19 SERPINB1A SERPINB10 ADAM8 F10 MMP9 SLPI ADAM33 HPSE F13A1
h
ECM Regulators
Cx3Cr1 Het vs. WT – 2mo
Cx3Cr1 KO vs. WT – 2mo
Fold Change
Adam8 Sulf1 Mmp25 F13a1 A2m Mmp9 Loxl3 Hpse Serpinb1a Serpinb8 Slpi F9 Itih5 Elane Serpinh1 Timp3 Ctso Mmp8 Ctsa Serpina3g Ctsb Cst3 Cstb Ctss Ctsc Egln1 Ogfod1 Tgm2 Fam20c Mmp14 Serpinf1 Adamts1 Htra3
i
WT – 1yr
KO – 1yr
46.6%
35.6%
17.8%

Supplementary Figure 5). Comparison of wild-type and Cx3Cr1-deficient microglia. **a)** 2-dimensional Sholl analysis was performed on ventral tegmental area (VTA) and nucleus accumbens (NAc) IBA1-positive microglia in young-adult (4 months; n=12 mice) and late-middle-aged (18 months; n=12 mice) mice. **b)** Sholl intersection plots for VTA and NAc microglia for young-adult and late-middle-aged mice. Error bars represent mean values +/- SEM. **c)** Example photomicrographs of GFP-positive microglia in the VTA of 12-month-old $Cx3Cr1^{EGFP/+}$(het) and $Cx3Cr1^{EGFP/EGFP}$ (KO) mice. **d)** Sholl intersection plots for VTA microglia from young-adult (3-4 months; n=4 het and n=4 hom mice) and early-middle-aged (12-15 months; n=4 het and n=4 hom mice). **e)** Fold changes of structural matrisome genes that were significantly differentially expressed between 1-year and 2-month wild-type (black) and Cx3Cr1-knockout (grey) microglia (from Gyoneva et al., 2019). **f)** Fold changes of structural matrisome genes that were significantly differentially expressed between 2-month Cx3Cr1 heterozygous (black) and Cx3Cr1-knockout (grey) microglia. **g)** Fold changes of regulatory matrisome genes that were significantly differentially expressed between 1 year and 2 month wild-type (black) and Cx3Cr1-knockout (grey) microglia. **h)** Fold changes of regulatory matrisome genes that were significantly differentially expressed between 2-month Cx3Cr1 heterozygous (black) and Cx3Cr1-knockout (grey) microglia. **i)** Venn diagram depicting the proportion of matrisome-related genes that were uniquely impacted by age in wild-type and Cx3Cr1-knockout microglia (top). Source data are provided in the file **Source Data - Figure S5.** * denotes p<0.05; ** denotes p<0.01.

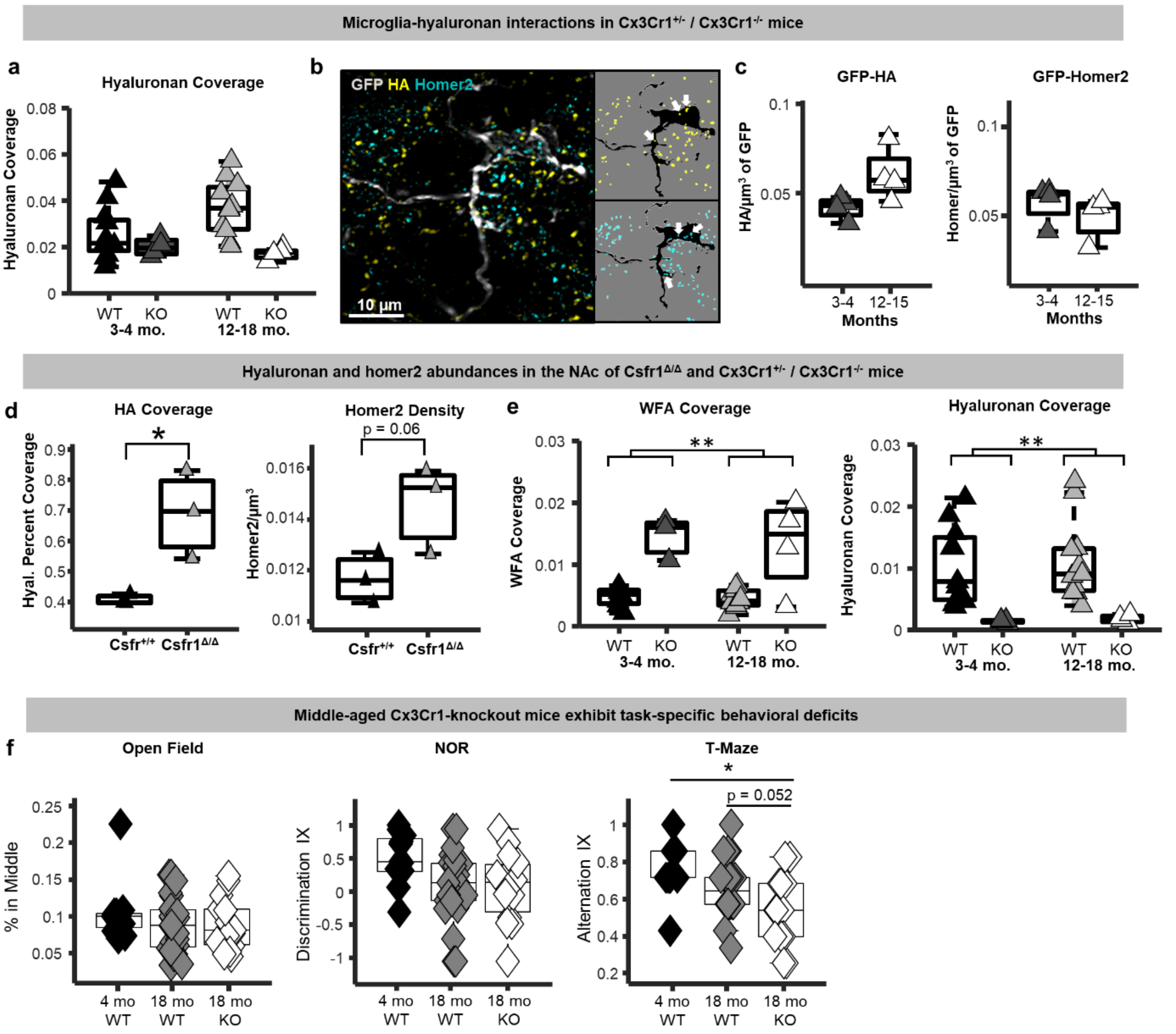

Supplementary Figure 6. ECM and behavioral properties in Cx3Cr1-deficient mice **a)** VTA hyaluronan tissue coverage in young-adult (3-4 months; n=12 wild type and n=4 Cx3Cr1 Cx3Cr1$^{EGFP/EGFP}$ mice) and middle-aged (12-18 months; n=12 wild type and n=4 Cx3Cr1 Cx3Cr1$^{EGFP/EGFP}$ mice) wild-type and Cx3Cr1-knockout (Cx3Cr1$^{EGFP/EGFP}$) mice. **b)** Putative microglia engulfment of hyaluronan and homer2 was also examined in Cx3Cr1$^{EGFP/EGFP}$ mice. **c)** Putative microglia-hyaluronan engulfment and microglia-homer2 engulfment in Cx3Cr1$^{EGFP/EGFP}$ mice. **d)** NAc hyaluronan tissue coverage and homer2 puncta densities in Csf1r$^{+/+}$ (black triangles; n=3 mice) and Csf1r$^{\Delta FIRE/\Delta FIRE}$ (grey triangles; n=3 mice) mice. **e)** WFA and hyaluronan tissue coverage in the NAc of young-adult (3-4 months) and middle-aged (12-18 months) wild-type and Cx3Cr1-knockout (Cx3Cr1$^{EGFP/EGFP}$) mice. **f)** Performance of young-adult (4 months) wild-type (n=12), middle-aged wild-type (18 months; n=24), and middle-aged (18 months) Cx3Cr1-knockout mice (n=15 mice) on an open field test, a novel object recognition test, and a T-Maze test. In all boxplots, boxes represent the interquartile range (IQR; 25-75 percentiles), the middle line represents the median, and whiskers extend +/- 1.5*IQR. Source data are provided in the file **Source Data - Figure S6.** * denotes p<0.05; ** denotes p<0.01.

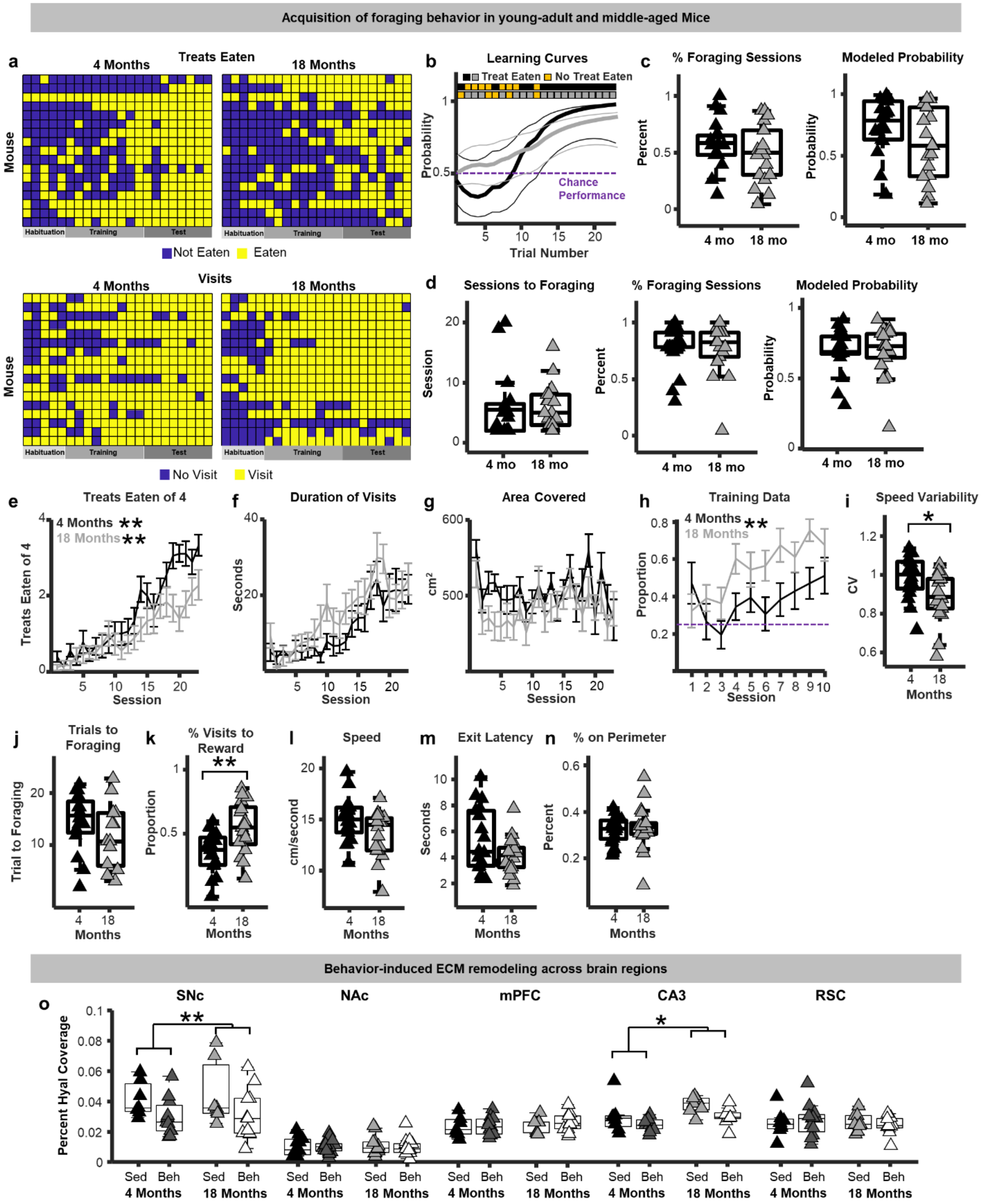

Supplementary Figure 7. Acquisition of foraging behaviors. **a)** Behavioral raster plots of treats eaten (top) and feeder visits (bottom) during encoding across sessions. **b)** Representative learning curves from hidden Markov modeling for a young-adult and late-middle-aged mouse. **c)** The proportion of sessions mice exhibited foraging behavior (ate at least 1 treat), and modeled estimates of the probability of foraging using **c)** treat-eaten and **d)** feeder visits data for young-adult (4 months; black) and late-middle-

aged (18 months; grey) mice. **e)** The number of treats eaten (of 4) across all encoding sessions for young-adult and middle-aged mice. **f)** The duration of feeder visits across sessions. **g)** The area covered across sessions for young-adult and late-middle-aged mice. **h)** The proportion of feeder visits to the correct feeder location during training sessions for young-adult and late-middle-aged mice. **i)** Variability in running speeds for young-adult and late-middle-aged mice. **j)** The number of trials to consistent foraging behavior. **k)** The proportion of rewarded feeder visits across training sessions. **l)** Average running speed, **m)** the latency to exit the start box, and **n)** proportion of time on the perimeter of the arena. **o)** Boxplots depicting hyaluronan (hyal) tissue coverage in young-adult sedentary (black; n=8 mice) and behavior-trained (dark grey; n=12 mice) mice and late-middle-aged sedentary (light grey; n=8 mice) and behavior-trained mice (white; n=12 mice) in substantia nigra pars compacta (SNc), nucleus accumbens (NAc)**,** medial prefrontal cortex (mPFC), CA3 region of the hippocampus, and retrosplenial cortex (RSC). Boxes in all boxplots represent the interquartile range (IQR; 25-75 percentiles), the middle line represents the median, and whiskers extend +/- 1.5*IQR. Source data are provided in the file **Source Data - Figure S7**. * denotes $p<0.05$; ** denotes $p<0.01$.

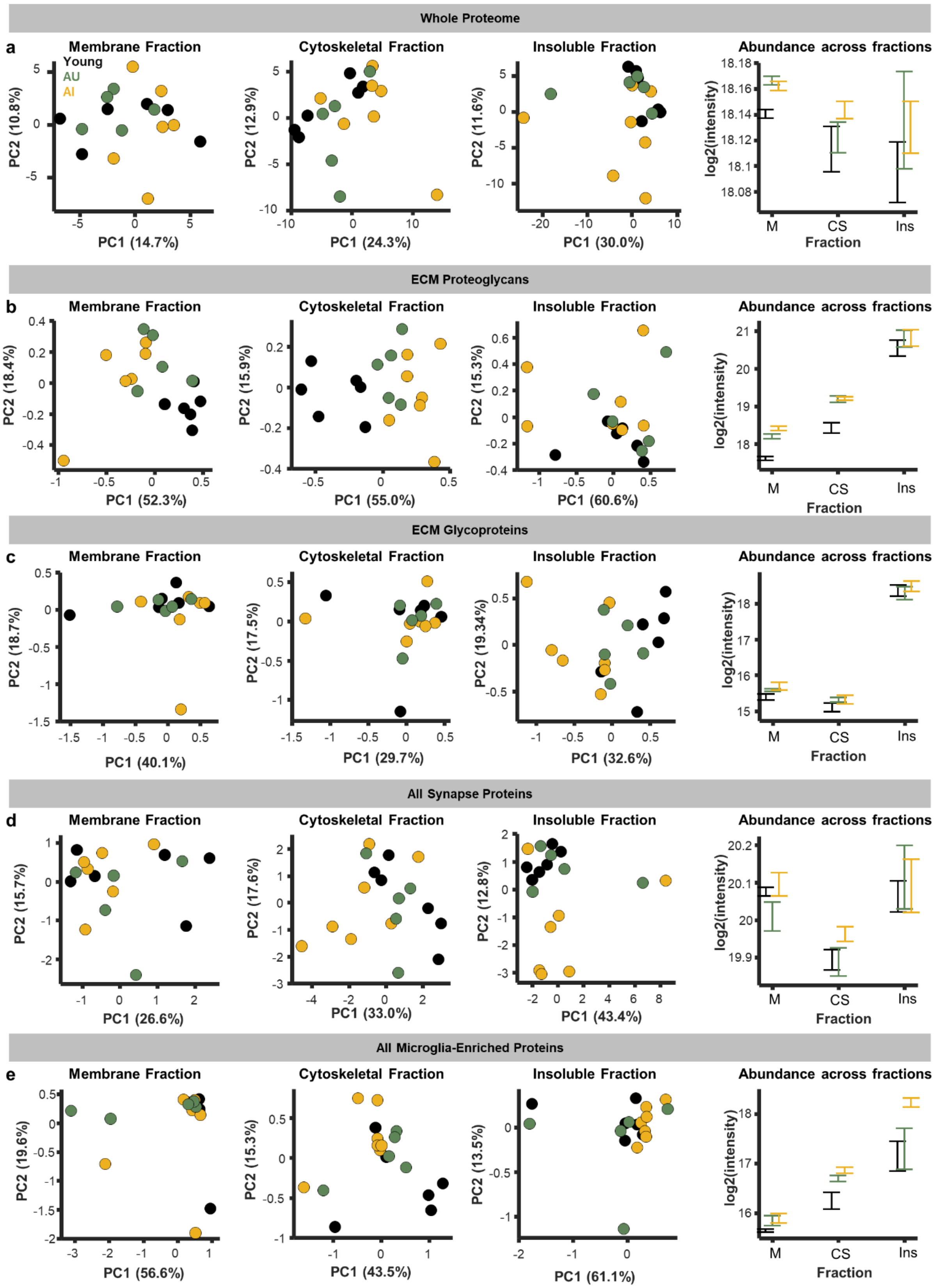

Whole Proteome
a
Membrane Fraction
Young
AU
AI
PC2 (10.8%)
PC1 (14.7%)
Cytoskeletal Fraction
PC2 (12.9%)
PC1 (24.3%)
Insoluble Fraction
PC2 (11.6%)
PC1 (30.0%)
Abundance across fractions
log2(intensity)
M
CS
Ins
Fraction
ECM Proteoglycans
b
Membrane Fraction
PC2 (18.4%)
PC1 (52.3%)
Cytoskeletal Fraction
PC2 (15.9%)
PC1 (55.0%)
Insoluble Fraction
PC2 (15.3%)
PC1 (60.6%)
Abundance across fractions
log2(intensity)
M
CS
Ins
Fraction
ECM Glycoproteins
c
Membrane Fraction
PC2 (18.7%)
PC1 (40.1%)
Cytoskeletal Fraction
PC2 (17.5%)
PC1 (29.7%)
Insoluble Fraction
PC2 (19.34%)
PC1 (32.6%)
Abundance across fractions
log2(intensity)
M
CS
Ins
Fraction
All Synapse Proteins
d
Membrane Fraction
PC2 (15.7%)
PC1 (26.6%)
Cytoskeletal Fraction
PC2 (17.6%)
PC1 (33.0%)
Insoluble Fraction
PC2 (12.8%)
PC1 (43.4%)
Abundance across fractions
log2(intensity)
M
CS
Ins
Fraction
All Microglia-Enriched Proteins
e
Membrane Fraction
PC2 (19.6%)
PC1 (56.6%)
Cytoskeletal Fraction
PC2 (15.3%)
PC1 (43.5%)
Insoluble Fraction
PC2 (13.5%)
PC1 (61.1%)
Abundance across fractions
log2(intensity)
M
CS
Ins
Fraction

Supplementary Figure 8. PCA analysis of different protein classes from tissue proteomic data in behaviorally-characterized mice. **a)** PCA plots of whole proteomes from membrane, cytoskeletal, and insoluble fractions (left 3 panels), and average log2-transformed protein intensities of the whole proteome across subcellular fractions. **b)** PCA plots of ECM proteoglycans from membrane, cytoskeletal, and insoluble fractions (left 3 panels), and average log2-transformed protein intensities of ECM proteoglycans across fractions. **c)** PCA plots of ECM glycoproteins from membrane, cytoskeletal, and insoluble fractions (left 3 panels), and average log2-transformed protein intensities of ECM glycoproteins across subcellular fractions. **d)** PCA plots of synapse proteins from membrane, cytoskeletal, and insoluble fractions (left 3 panels), and average log2-transformed protein intensities of synapse proteins across subcellular fractions. **e)** PCA plots of all microglia-enriched proteins from membrane, cytoskeletal, and insoluble fractions (left 3 panels), and average log2-transformed protein intensities of microglia-enriched proteins across subcellular fractions. All data from n=6 young, n=5 middle-aged unimpaired, and n=7 middle-aged impaired mice. Source data are provided in the file **Source Data - Figure S8.**

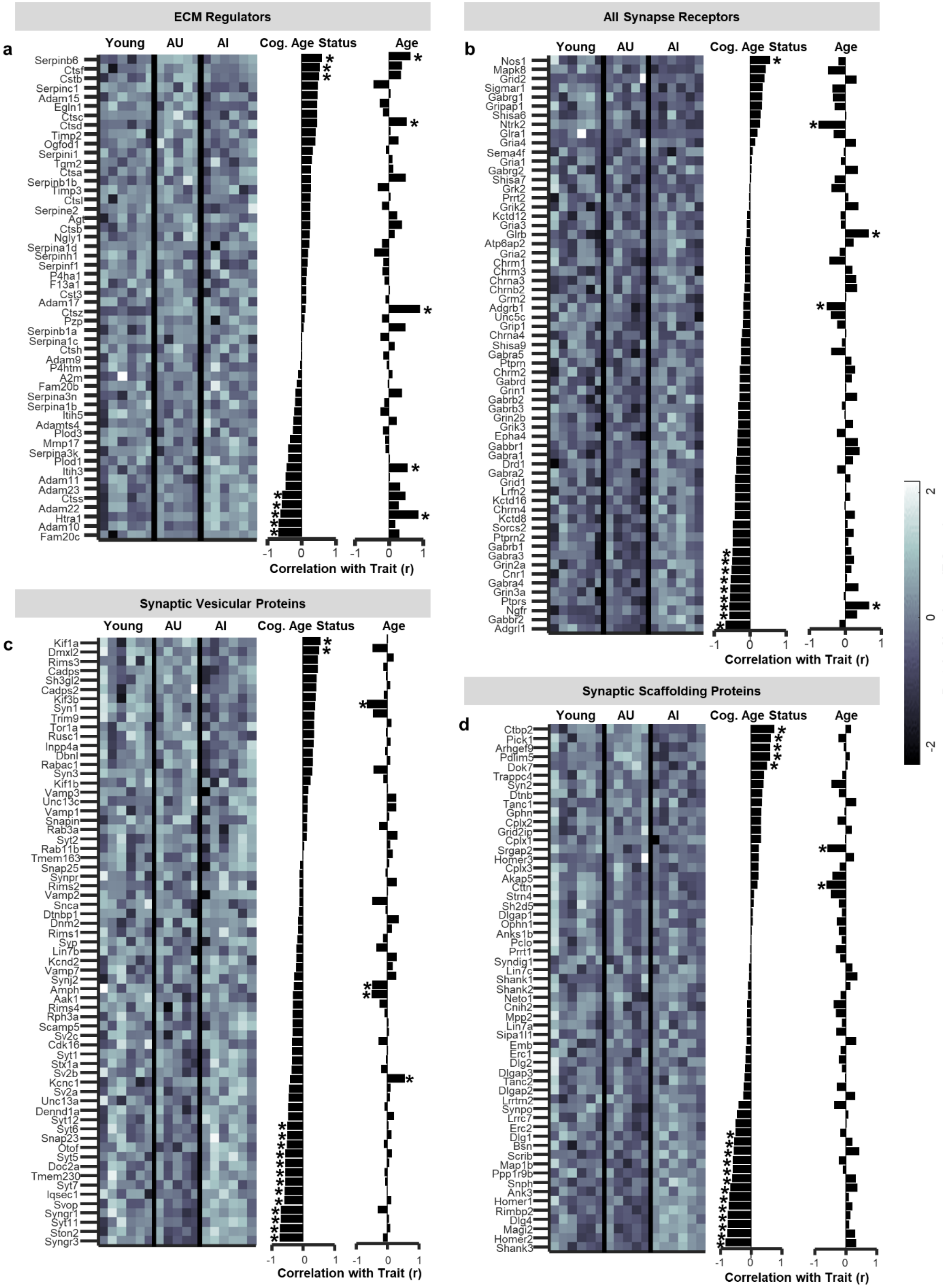

ECM Regulators
a
Young
AU
AI
Cog. Age Status
Age
Serpinb6
Ctsf
Cstb
Serpinc1
Adam15
Egln1
Ctsc
Ctsd
Timp2
Ogfod1
Serpini1
Tgm2
Ctsa
Serpinb1b
Timp3
Ctsl
Serpine2
Agt
Ctsb
Ngly1
Serpina1d
Serpinh1
Serpinf1
P4ha1
F13a1
Cst3
Adam17
Ctsz
Pzp
Serpinb1a
Serpina1c
Ctsh
Adam9
P4htm
A2m
Fam20b
Serpina3n
Serpina1b
Itih5
Adamts4
Plod3
Mmp17
Serpina3k
Plod1
Itih3
Adam11
Adam23
Ctss
Adam22
Htra1
Adam10
Fam20c
-1 0 1
-1 0 1
Correlation with Trait (r)
All Synapse Receptors
b
Young
AU
AI
Cog. Age Status
Age
Nos1
Mapk8
Grid2
Sigmar1
Gabrg1
Gripap1
Shisa6
Ntrk2
Glra1
Gria4
Sema4f
Gria1
Gabrg2
Shisa7
Grk2
Prrt2
Grik2
Kctd12
Gria3
Glrb
Atp6ap2
Gria2
Chrm1
Chrm3
Chrna3
Chrnb2
Grm2
Adgrb1
Unc5c
Grip1
Chrna4
Shisa9
Gabra5
Ptprn
Chrm2
Gabrd
Grin1
Gabrb2
Gabrb3
Grin2b
Grik3
Epha4
Gabbr1
Gabra1
Drd1
Gabra2
Grid1
Lrfn2
Kctd16
Chrm4
Kctd8
Sorcs2
Ptprn2
Gabrb1
Gabra3
Grin2a
Cnr1
Gabra4
Grin3a
Ptprs
Ngfr
Gabbr2
Adgrl1
-1 0 1
-1 0 1
Correlation with Trait (r)
Synaptic Vesicular Proteins
c
Young
AU
AI
Cog. Age Status
Age
Kif1a
Dmxl2
Rims3
Cadps
Sh3gl2
Cadps2
Kif3b
Syn1
Trim9
Tor1a
Rusc1
Inpp4a
Dbnl
Rabac1
Syn3
Kif1b
Vamp3
Unc13c
Vamp1
Snapin
Rab3a
Syt2
Rab11b
Tmem163
Snap25
Synpr
Rims2
Vamp2
Snca
Dtnbp1
Dnm2
Rims1
Syp
Lin7b
Kcnd2
Vamp7
Synj2
Amph
Aak1
Rims4
Rph3a
Scamp5
Sv2c
Cdk16
Syt1
Stx1a
Sv2b
Kcnc1
Sv2a
Unc13a
Dennd1a
Syt12
Syt6
Snap23
Otof
Syt5
Doc2a
Tmem230
Syt7
Iqsec1
Svop
Syngr1
Syt11
Ston2
Syngr3
-1 0 1
-1 0 1
Correlation with Trait (r)
Synaptic Scaffolding Proteins
d
Young
AU
AI
Cog. Age Status
Age
Ctbp2
Pick1
Arhgef9
Pdlim5
Dok7
Trappc4
Syn2
Dtnb
Tanc1
Gphn
Cplx2
Grid2ip
Cplx1
Srgap2
Homer3
Cplx3
Akap5
Cttn
Strn4
Sh2d5
Dlgap1
Ophn1
Anks1b
Pclo
Prrt1
Syndig1
Lin7c
Shank1
Shank2
Neto1
Cnih2
Mpp2
Lin7a
Sipa1l1
Emb
Erc1
Dlg2
Dlgap3
Tanc2
Dlgap2
Lrrtm2
Synpo
Lrrc7
Erc2
Dlg1
Bsn
Scrib
Map1b
Ppp1r9b
Snph
Ank3
Homer1
Rimbp2
Dlg4
Magi2
Homer2
Shank3
-1 0 1
-1 0 1
Correlation with Trait (r)
Protein Abundance (Z Score)
2
0
-2

Supplementary Figure 9. Relationships of synapse protein abundances with age and cognitive status. **a)** Heat plot of all detected ECM regulatory protein abundances and bar plots of their relationship with cognitive status and age (* $p<0.05$; linear probability model). **b)** Heat plot of all detected excitatory, inhibitory, and neuromodulator receptor abundances and bar plots of their relationship with cognitive status and age (* $p<0.05$; linear probability model). **c)** Heat plot of all detected synaptic vesicle proteins abundances and bar plots of their relationship with cognitive status and age (* $p<0.05$; linear probability model). **d)** Heat plot of all detected synaptic scaffolding proteins abundances and bar plots of their relationship with cognitive status and age (* $p<0.05$; linear probability model). All data from n=6 young, n=5 middle-aged unimpaired, and n=7 middle-aged impaired mice. Source data are provided in the file **Source Data - Figure S9**.

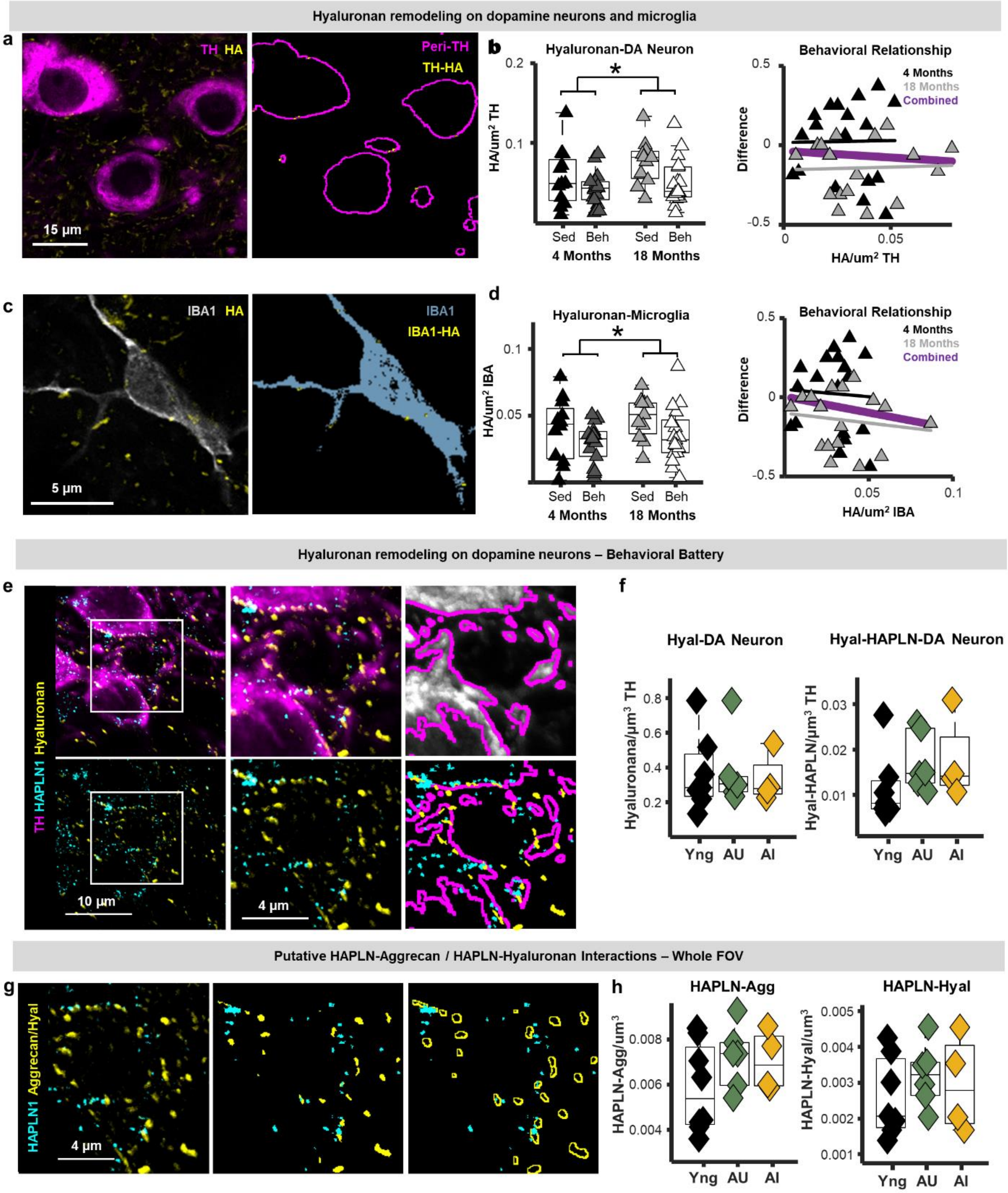

Supplementary Figure 10. Hyaluronan, hyaluronan and proteoglycan link protein 1 (HAPLN1), and aggrecan proximity analysis results**. a)** Left: photomicrograph of histochemically labelled tyrosine hydroxylase (TH)-positive neurons and hyaluronan in the VTA of a middle-aged mouse. Right: peri-TH ROIs (magenta) used to index hyaluronan fragment on dopamine neurons (hyaluronan-DA neuron; yellow). **b)** Left: Boxplots depicting hyaluronan-DA neuron fibril densities normalized to TH in young-adult sedentary (black; n=12 mice) and behavior-trained (dark grey; n=18 mice) mice and late-middle-aged sedentary (light grey; n=12 mice) and behavior-trained mice (white; n=18 mice). Right: Relationship between TH-hyaluronan fibril densities and performance on reward-based foraging task. **c)** Left:

photomicrograph of an IBA1-positive microglia cell body and proximal branches and hyaluronan in the VTA of a middle-aged mouse. Right: IBA1 ROI used to examine hyaluronan fragments in contact with microglia (hyaluronan-microglia). **d)** Left: Boxplots depicting hyaluronan-microglia fibril densities normalized to IBA1. Right: Relationship between IBA1-hyaluronan fibril densities and performance on reward-based foraging task. **e)** Example photomicrographs of histochemically labelled hyaluronan, HAPLN1, and tyrosine hydroxylase (TH) from the VTA of a late-middle aged mouse. The middle panels depict the fields of view depicted by the white squares in the left panel. Right: example of the peri-dopamine neuron region of interest used to estimate the abundance of hyaluronan and HAPLN1 on dopamine neuron surfaces in the VTA. **f)** Boxplots depicting hyaluronan-DA neuron puncta densities (left) and hyaluronan-HAPLN1-DA neuron puncta densities (right) in the VTA of young (black; n=8 mice), aging unimpaired (AU; green; n=6 mice), and aging impaired (AI; purple; n=4 mice) mice. **g)** Schematic of hyaluronan-HAPLN and aggrecan-HAPLN1 proximity analysis to estimate the density of hyaluronan fibrils <0.5 microns from HAPLN1. Note: this example photomicrograph is the same as in **e**. **h)** Boxplots depicting aggrecan-HAPLN1 (left) and hyaluronan-HAPLN1 (right) puncta densities (left) in the VTA of young, aging unimpaired (AU), and aging impaired (AI) mice. In all boxplots, boxes represent the interquartile range (IQR; 25-75 percentiles), the middle line represents the median, and whiskers extend +/- 1.5*IQR. Source data are provided in the file **Source Data - Figure S10.** * denotes $p<0.05$
