# Supplementary material for: Midbrain extracellular matrix and microglia are associated with cognition in aging mice
Source: Nat Commun. 2025 Nov 27;16:11319. doi: 10.1038/s41467-025-66434-z (PMC12722224; doi:10.1038/s41467-025-66434-z)
Supplement: Supplementary file 2 — Description of Additional Supplementary Files [file 41467_2025_66434_MOESM2_ESM.pdf]

Description of Additional Supplementary Files

Supplementary Data 1 | Log2-transformed protein intensity values from comparison of ECM e nrichment methods (solubility-based fractionation vs. chaotropic extraction and digestion; Ext. Data Figure 1).

Supplementary Data 2 | Log2-transformed protein intensity values of midbrain and striatum samples from young-adult and aged mice (Figure 1). Data from each solubility fraction (cytosolic, nuclear, membrane, cytoskeletal, and insoluble) are provided separately for each region.

Supplementary Data 3 | MS metrics for proteomic data in Figure 1, as well as metadata information.

Supplementary Data 4 | Log2-transformed protein intensity values from quantitative proteomic experiments of the midbrain of behaviorally-characterized mice (Figure 6). Data from membrane, cytoskeletal, and insoluble fractions are provided separately.

Supplementary Data 5 | MS metrics collected for each sample in Figure 6 (midbrain proteomes of behaviorally characterized aging mice), as well as metadata information.

Supplementary Data 6 | Source statistics for all main figures.
