# Supplementary material for: Midbrain extracellular matrix and microglia are associated with cognition in aging mice
Source: Nat Commun. 2025 Nov 27;16:11319. doi: 10.1038/s41467-025-66434-z (PMC12722224; doi:10.1038/s41467-025-66434-z)
Supplement: Supplementary file 9 — Reporting Summary [file 41467_2025_66434_MOESM9_ESM.pdf]

nature portfolio

Corresponding author(s): Daniel Gray

Last updated by author(s): Oct 14, 2025

Reporting Summary

Nature Portfolio wishes to improve the reproducibility of the work that we publish. This form provides structure for consistency and transparency in reporting. For further information on Nature Portfolio policies, see our Editorial Policies and the Editorial Policy Checklist.

Statistics

For all statistical analyses, confirm that the following items are present in the figure legend, table legend, main text, or Methods section.

n/a	Confirmed	
☐	☒	The exact sample size (*n*) for each experimental group/condition, given as a discrete number and unit of measurement
☐	☒	A statement on whether measurements were taken from distinct samples or whether the same sample was measured repeatedly
☐	☒	The statistical test(s) used AND whether they are one- or two-sided<br>*Only common tests should be described solely by name; describe more complex techniques in the Methods section.*
☐	☒	A description of all covariates tested
☐	☒	A description of any assumptions or corrections, such as tests of normality and adjustment for multiple comparisons
☐	☒	A full description of the statistical parameters including central tendency (e.g. means) or other basic estimates (e.g. regression coefficient) AND variation (e.g. standard deviation) or associated estimates of uncertainty (e.g. confidence intervals)
☐	☒	For null hypothesis testing, the test statistic (e.g. *F*, *t*, *r*) with confidence intervals, effect sizes, degrees of freedom and *P* value noted<br>*Give P values as exact values whenever suitable.*
☒	☐	For Bayesian analysis, information on the choice of priors and Markov chain Monte Carlo settings
☒	☐	For hierarchical and complex designs, identification of the appropriate level for tests and full reporting of outcomes
☒	☐	Estimates of effect sizes (e.g. Cohen's *d*, Pearson's *r*), indicating how they were calculated

Our web collection on statistics for biologists contains articles on many of the points above.

Software and code

Policy information about availability of computer code

Data collection	Confocal Microscopy: Zen 2 Software (Zeiss); LAS X Software (Leica)<br>Behavioral Tracking: Bioserve Viewer3 Software (Bioserve)<br>Proteomics:Maxquant software (Max Planck Institute of Biochemistry)
Data analysis	ImageJ-win32 1.54f (NIH)<br>RStudio 2021.09.2 (Posit)<br>WGCNA (R Package)<br>Matlab; Version 9.11 2021b (MathWorks)<br>Bioinfomatics ToolBox; Version 4.15.2 (Matlab toolbox)<br>Image Processing Toolbox; Version 11.4 (Matlab Toolbox)<br>Statistics and Machine Learning Toolbox; Version 12.2 (Matlab Toolbox)

For manuscripts utilizing custom algorithms or software that are central to the research but not yet described in published literature, software must be made available to editors and reviewers. We strongly encourage code deposition in a community repository (e.g. GitHub). See the Nature Portfolio guidelines for submitting code & software for further information.

Data

Policy information about availability of data

All manuscripts must include a data availability statement. This statement should provide the following information, where applicable:

- Accession codes, unique identifiers, or web links for publicly available datasets
- A description of any restrictions on data availability
- For clinical datasets or third party data, please ensure that the statement adheres to our policy

Raw proteomics data is available on the MassIVE data repository under the accession key MSV000096508. Log2 transformed proteomics data, microscopy data, and behavioral data is provided in supplementary source data tables included with this manuscript. Raw microscopy images are available upon request from the authors.

Research involving human participants, their data, or biological material

Policy information about studies with human participants or human data. See also policy information about sex, gender (identity/presentation), and sexual orientation and race, ethnicity and racism.

Reporting on sex and gender	n/a
Reporting on race, ethnicity, or other socially relevant groupings	n/a
Population characteristics	n/a
Recruitment	n/a
Ethics oversight	n/a

Note that full information on the approval of the study protocol must also be provided in the manuscript.

Field-specific reporting

Please select the one below that is the best fit for your research. If you are not sure, read the appropriate sections before making your selection.

- [x] Life sciences
- [ ] Behavioural & social sciences
- [ ] Ecological, evolutionary & environmental sciences

For a reference copy of the document with all sections, see nature.com/documents/nr-reporting-summary-flat.pdf

Life sciences study design

All studies must disclose on these points even when the disclosure is negative.

Sample size	Sample sizes for behavioral experiments were determined using power analyses from preliminary data collected within the lab and from published data using similar tasks. No statistical methods were used to predetermine sample sized for histological and proteomic experiments, but sample sizes are consisted with data reported in previous publications.
Data exclusions	One outlier was detected in the WGCNA analysis and was removed. For all histological and behavioral data, outlier testing was performed using Grubb's test.
Replication	For tissue proteomic datasets, two distinct batches of young-adult and aging mice were used and results from these analyses are consistent with each other.<br><br>For histological experiments, 2 distinct cohorts of mice were used and results were also consistent.<br><br>For behavioral experiments, two distinct approaches using different cohorts of aging mice were used and both results detected age-related shifts in cognitive function.
Randomization	Mice from both sexes within a batch were randomly assigned to sedentary and behavioral conditions.<br><br>Mice for proteomic experiments were randomly selected from larger cohorts.
Blinding	All analyses were performed blind to the identity of the animals.

Reporting for specific materials, systems and methods

We require information from authors about some types of materials, experimental systems and methods used in many studies. Here, indicate whether each material, system or method listed is relevant to your study. If you are not sure if a list item applies to your research, read the appropriate section before selecting a response.

Materials & experimental systems

n/a	Involved in the study	
☐	☒	Antibodies
☒	☐	Eukaryotic cell lines
☒	☐	Palaeontology and archaeology
☐	☒	Animals and other organisms
☒	☐	Clinical data
☒	☐	Dual use research of concern
☒	☐	Plants

Methods

n/a	Involved in the study	
☒	☐	ChIP-seq
☒	☐	Flow cytometry
☒	☐	MRI-based neuroimaging

Antibodies

Antibodies used	biotinylated hyaluronan binding protein lectin (1:250; Sigma-Aldrich cat: 385911)<br>biotinylated Wisteria floribunda lectin (1:100; Vector Labs cat: B-1355)<br>goat anti-HAPLN1 (1:200; R & D Systems Cat: AF2608)<br>goat anti-GFP (1:1000; Frontier Institute cat: GFP-go-Af1480)<br>rabbit anti-aggrecan (1:200; Sigma Aldrich cat: AB1031)<br>rabbit-anti-IBA1 (1:500; Wako cat: 019-19741)<br>chicken anti tyrosine hydroxilase (1:500; Aves cat: TYH)<br>rabbit anti-Homer2 (1:2000; Synaptic Systems cat: 160 003)<br>mouse anti-Vglut1 (1:2000; DSBH cat: N28/9)
Validation	Antibodies were selected according to validation profiles reported by the distributing manufacturers and in publications:<br>biotinylated hyaluronan binding protein lectin: validated through in vitro binding assays and histochemistry following hyaluronidase injection (Soria et al., 2020).<br>biotinylated Wisteria floribunda lectin: This lectin is the most widely used marker of the ECM in neuroscience and has been included in at least 295 citations.<br>goat anti-HAPLN1: validated by manufacturer via affinity purification.<br>goat anti-GFP : validated by immunohistochemical staining of GFP-transgenic mice and wild-type mice.<br>rabbit-anti-IBA1: validated through immunohistochemical staining of microglia-depleted mice (Csf1rΔFIRE/ΔFIRE)<br>chicken anti tyrosine hydroxilase: This antibody has been used for IHC in at least 86 peer-reviewed publications.<br>rabbit anti-Homer2: validated by manufacturer via affinity purification.<br>mouse anti-Vglut1 was validated by manufacturer via western blot of mouse brain (positive band) and mouse spleen lysates (negative band).

Animals and other research organisms

Policy information about studies involving animals; ARRIVE guidelines recommended for reporting animal research, and Sex and Gender in Research

Laboratory animals	C57Bl6 wild-type mice (3-4 months and 18-22 months)<br>CX3CR1EGFP/EGFP mice (3-4 months and 12-15 months)<br>Csf1r+/+ and Csf1rΔFIRE/ΔFIRE mice (3-4 months).
Wild animals	n/a
Reporting on sex	Potential sex differences in behavioral, proteomic, and histological data were tested and statistics are available in Supplementary Table 6.
Field-collected samples	n/a
Ethics oversight	UCLA Animal Research Committee (ARC) and UCLA institutional animal care and use committee (IACUC).

Note that full information on the approval of the study protocol must also be provided in the manuscript.

Plants

Seed stocks	n/a
Novel plant genotypes	n/a
Authentication	n/a
